# Research Progress in the Medical Application of Heavy Water, Especially in the Field of D_2_O-Raman Spectroscopy

**DOI:** 10.7150/ijms.73150

**Published:** 2022-07-18

**Authors:** Xin Wang, Nai-meng Liu, Ya-fei Zhao, Fan Yang, Zi-jia Zhu, Dong Song

**Affiliations:** Department of Breast Surgery, First Hospital of Jilin University, Changchun, Jilin 130021

**Keywords:** heavy water, deuterium oxide, Raman microspectroscopy, isotypes, probe, medicine.

## Abstract

Heavy water is an ideal contrast agent for metabolic activity and can be adapted to a wide range of biological systems owing to its non-invasiveness, universal applicability, and cost-effectiveness. As a new type of probe, the heavy isotope of water has been widely used in the study of cell development, metabolism, tissue homeostasis, aging, and tumor heterogeneity. Herein, we review findings supporting the applications of and research on heavy water in monitoring of bacterial metabolism, rapid detection of drug sensitivity, identification of tumor cells, precision medicine, and evaluation of skin barrier function and promote the use of heavy water as a suitable marker for the development of detection and treatment methodologies.

## Introduction

**Figure 1:**
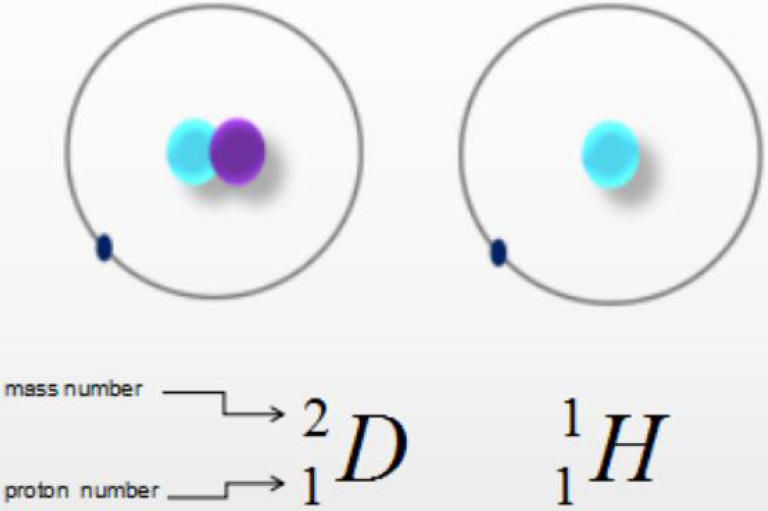
** Chemical formula.** Water is a compound of hydrogen (H) and oxygen (O), while heavy water is a compound of heavy hydrogen (D) and oxygen (O).

Heavy water, also known as deuterium oxide or deuterium water, is a compound of heavy hydrogen (D) and oxygen (O) (**Fig. 1**). A molecule of heavy water is composed of two heavy hydrogen and one oxygen atom, with the molecular and chemical formula D_2_O. Heavy water is similar to ordinary water in appearance but has a higher density of 1.1079 g/cm^3^, as well as higher freezing and boiling points of 3.82 °C and 101.42 °C, respectively. The relative molecular mass of heavy water, 20.0275 Mr, is higher than that of water (H_2_O, 18.0153 Mr) by about 11%, hence the name “heavy” water. The differences between deuterium and hydrogen are negligible; while they have different neutron numbers, mass numbers, and physical properties, they have the same number of protons, number of outermost electrons, and chemical properties. Hence, the chemical properties of heavy water and ordinary water are also very similar. It has been proved that a small amount of heavy water does not cause adverse reactions 1-2. Heavy water has been widely used as a probe in many fields.

## Quantitative determination of heavy water concentration

Currently, the commonly used methods to quantify heavy water concentration include the density method, mass spectrometry, and spectroscopy techniques 3. Among these, Raman spectroscopy has attracted increasing attention as a highly sensitive molecular imaging technique for studying complex biological systems, including cells, tissues, and various biological materials 4. Raman spectroscopy analyzes the biochemical components of a sample by measuring the inelastic scattering of light by different molecular species, producing a spectrum based on the chemical bonds present within the analyzed samples. This technique allows non-destructive, label-free spectral imaging and analysis of cells, tissues, and nanoparticles 5-7. As a new quantitative method, heavy water-labeled single-cell Raman microspectroscopy can reduce damage to cells and human interference factors. This method does not alter the cell composition or rely on cell culture and thus allows the rapid, quantitative, and nondestructive evaluation of the effect of drugs on the real-time metabolic activity of microorganisms at the single-cell level 8-10. Simultaneously, the results of Raman spectroscopy provide abundant information, which can reflect the biochemical and structural characteristics of the material of interest. In theory, this method can detect the entire spectrum of substances in the cell, especially the “fingerprint area” in the range of ~600-1800 cm^-1^, which can distinguish cell types and explore the mechanism of microbial stress 11-13. Thus, this technique has a broad application prospect and important guiding significance.

**Figure 2:**
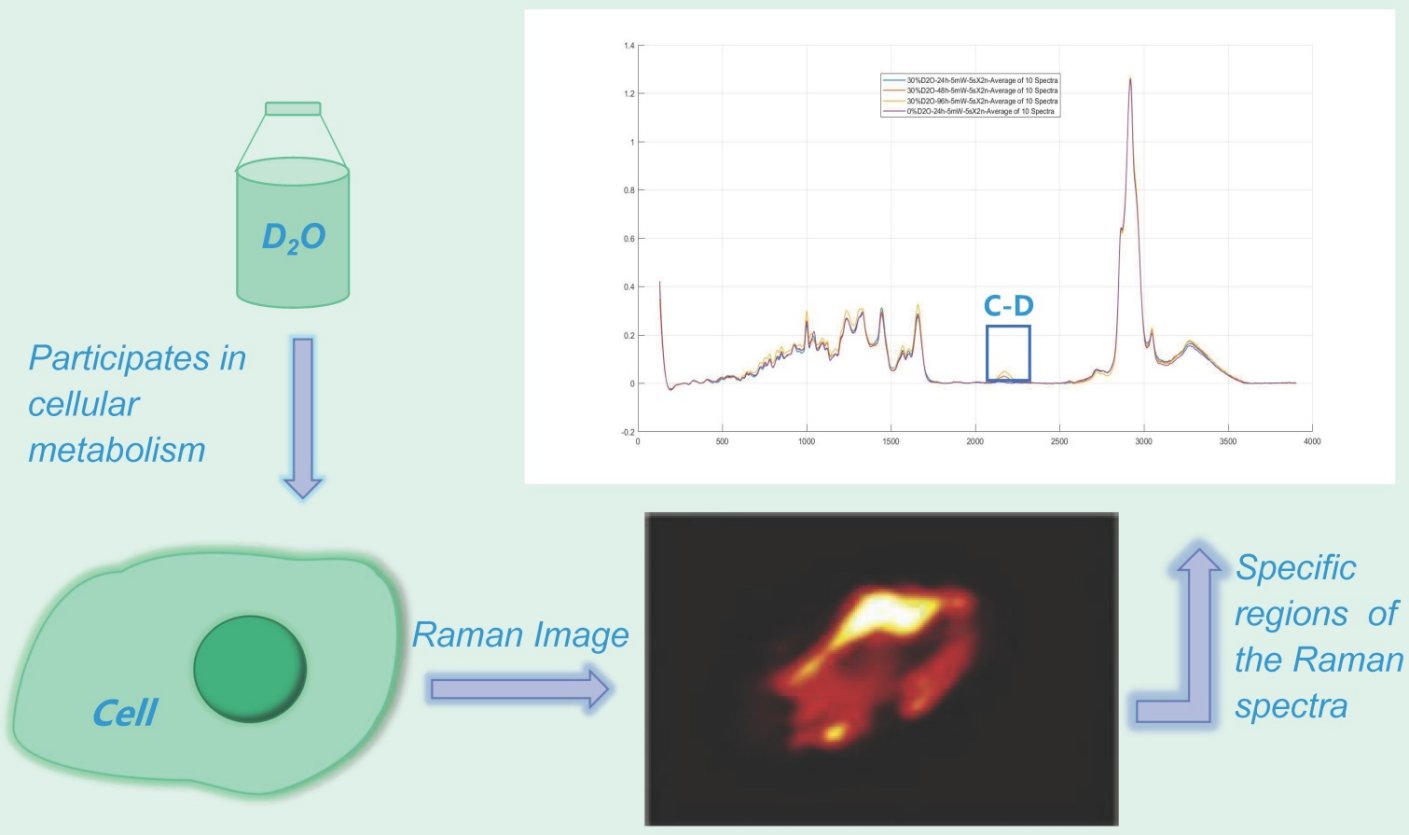
** Heavy water Raman spectroscopy imaging.** The heavy water peak (C-D peak) can be measured in the specific region of the Raman spectrum (~ 2040-2300 cm^-1^) by using a certain concentration of heavy-water cultured microorganisms.

The presence of heavy water results in heavy water peaks (C-D peaks) in specific regions (~2040-2300 cm^-1^) of the Raman spectra of metabolically active microorganisms (**Fig. 2**) 14-15, which correspond to symmetric and asymmetric C-D stretching vibrations caused primarily by lipids and proteins 12,16-17. Furthermore, the minimum inhibitory concentration based on metabolic activity (MIC-MA) can be used to quantitatively evaluate the effect of drugs on real-time metabolic activity of microorganisms based on the ΔC-D ratio (the difference between the current time and the baseline C-D) 8-9. This technology has been widely used in microbial species identification and has also been widely used to evaluate the effect of drugs on bacterial metabolic activity.

## Biosafety of heavy water

Compared with other isotope markers, heavy water exerts little effect on microorganisms; furthermore, it is cost effective. The growth of bacteria and single-cell fungi in heavy water within a certain concentration range was not significantly inhibited (P > 0.05) compared with that in normal water 8. Prior studies have suggested that cells and single-celled fungi can tolerate a certain concentration of heavy water, and thus, heavy water toxicity is not a limiting factor in this technique. In mice, a concentration of heavy water below 20% had no effect on physiological processes or cell division; did not modulate physiology, growth, appetite, or reproduction; and was found to have no teratogenic effect in multi-generation studies 18-21. Studies have found that drinking 60-70 ml of heavy water daily would not cause adverse reactions 23-24. Therefore, deuterium has been widely used as a stable isotope to evaluate the human body composition and metabolic rate 19,24-25.

The determination of heavy water concentration was used for rapid evaluation of the effect of drugs on the metabolic activity of cells and single-cell fungi, with high sensitivity 26. The labeling of heavy water as a probe has been widely used environmental studies. Li et al. used the heavy water labeling method to understand the metabolic flux of microbial communities in complex soil systems. Through heavy water modification of soil microorganisms, bacteria that release phosphate were identified according to the ratio of C-D stretching vibration to Raman spectroscopy 27. Eichorst et al. also found the same method of heavy water labeling, which was more conducive to the objective evaluation of differences in metabolic activity of soil bacteria and proved that deuterium content was suitable for the detection of metabolic activity indicators 28. A recent study by Taubert et al., using heavy water as a probe in the study of groundwater microbial communities, proved that this probe could identify active microorganisms in groundwater and their functional characteristics 29. The special value of heavy water is reflected in the application of atomic energy technology. Heavy water reduces neutron velocity and controls nuclear fission by acting as a retarder in nuclear reactors.

## Medical applications of heavy water in the field of D_2_O-Raman spectroscopy

### Monitoring metabolism in individual bacteria

Using heavy water with stable isotope labeling, deuterium intake was found to be a reliable indicator of general bacterial metabolic activity 30-31. Because neither H_2_O nor D_2_O have Raman peaks in the C-D region, the background from water did not interfere with this approach 32. Therefore, studies have been established to examine the effects of carbon sources and bacteria on deuterium uptake by quantitatively measuring the assimilation of heavy water into a single bacterium 33-34. The deuterium assimilation rate was higher in the presence of simple substrates, such as sugar, compared to that with complex carbon substrates, and the difference was significant in bacterial isolates. The quantitative determination of deuterium content in heavy water was further used to distinguish between various types of bacteria and their metabolic activities; thus, this detection method could be combined with chemometrics to construct a powerful bacterial monitoring method. The absorption of deuterium as a marker of bacterial metabolic activity determined using Raman microspectroscopy was strongly affected by the organic carbon source used by a single bacterium for growth, as well as by the cell itself 35.

### Rapid detection technology of drug sensitivity

To counteract the common problem of antibiotic resistance 36-37, it is urgent that researchers develop technology to rapidly detect antibiotic drug sensitivity in clinic 38-39. As metabolism suppressive drugs ultimately alter cellular macromolecule metabolism, a method for detecting macromolecule-specific metabolites following drug therapy would be valuable. The application of heavy water as a probe detection method and the C-D band shift as a biomarker of cellular metabolic activity to quantitatively evaluate the metabolic activity of bacteria in their environment in a culture-independent manner at the single-cell level has been proposed to determine the efficacy of antibiotics through metabolic inhibition 30. In particular, the ability to evaluate specific metabolites and newly synthesized macromolecules provides greater insights into the underlying processes by which cells respond to drugs. Using heavy water as a probe to detect low numbers of bacteria solved the clinical problem of low sample content. By combining single-cell Raman spectroscopy and heavy water labeling, the active response of bacteria to antibiotics can be evaluated according to the deuterium-related characteristic peaks after only 30 minutes 40. By evaluating the differences in heavy water assimilation activity between drug-resistant bacteria and sensitive bacteria under the action of antibiotics, the total time from urine collection to drug sensitivity reading can be reduced to 2.5 hours, which can guide clinicians to perform rapid diagnosis and screen effective antibiotics in time 41. Therefore, this method can be the basis of a new antimicrobial screening platform at the single-cell level.

### Identification of tumor stem cells

Fast-growing tumor cells also appear to incorporate more D from heavy water compared to other cells; this property would be detected directly with a Raman microscope. Using heavy water in combination with Raman microscopy, the boundaries of a tumor can be revealed by its inherently higher metabolic activities compared to the surrounding normal tissue. Thus, Raman spectroscopy using heavy water as a probe can help identify tumor stem cells with specific patterns of metabolic activity 42-44. Studies have found that unsaturated lipid levels in ovarian cancer stem cells increased significantly compared to those in normal cells 45-46. In addition, the combination of heavy water-Raman spectroscopy and fluorescence labeling can be used to monitor the metabolic activity of specific cells and lineages *in situ*, especially the metabolic cooperation between glial cells and neurons 47. In addition to monitoring the synthesis of lipids and proteins 48, heavy water-Raman spectroscopy can be used to monitor protein turnover, lipid consumption, and macromolecular degradation in tumor cells 49.

### Positioning of precise targeted drug use

Extracellular vesicles are biologically derived nanocarriers important for intercellular communication and transportation that have been proposed as disease biomarkers and therapeutic drug carriers 50-51. The combination of heavy water-Raman spectroscopy imaging and bioactive molecules provides an opportunity to study the production and uptake of extracellular vesicles in various normal and dysfunctional states, as well as a direction for precise drug therapy 52-53. Applying this approach to various newly designed drugs that target cell metabolism can identify which macromolecules are specifically targeted by the drug. This method can produce information-rich whole-cell spectral data and can be used to directly visualize and analyze extracellular vesicles at the two- and three-dimensional levels, thus providing guidance for the design of future extracellular vesicle treatment systems 54-55.

### Assessment of skin barrier function

Heavy water can be used as an excellent and cost-effective probe to evaluate skin barrier function. The penetration dynamics of water can be regulated by the integrity of the skin barrier 56-57. Therefore, the application of D_2_O is a promising method to assess the state of the skin barrier, considering the isotope substitution and diffusion behavior of water 58-59. Owing to the different Raman spectral characteristics of the O-D bond of heavy water and the O-H bond of the skin, the influence of external osmotic water can be minimized 60-61. The combination of heavy water and Raman microscopy can sensitively identify small changes in the molecular composition of skin and can detect skin at different depths. Heavy water, as a skin probe, can be used to detect skin water-related properties by extracting spectra from each pixel depth and distinguishing endogenous and exogenous hydrogen bonds 62-63. By using different hydrogen-bonding water types to calculate the relative water content, the total water content of different skin depths can be calculated 64.

## Other medical applications of heavy water

Deuterium in water has been reported to be rapidly balanced with mediators such as urine, saliva or serum 65-67, which can be used to measure the total amount of water in the body 68. Lichtenbelt et al. showed that a 10-hour sampling time appears to be preferable for measuring total body water space and body composition by the deuterium-dilution technique 69. Studies have shown that D_2_O can be used as a tracer to measure tissue perfusion and blood flow 70-71. Recently, Lin Chen et al. demonstrated that D_2_O can be used as a new contrast agent to guide intravascular neurointervention and that deuterium-based MRI is a secure and practical method that can to accurately identify the perfusion area and to predict the affected area, which can guide real-time endovascular intervention 72. Studies have demonstrated the potential of the deuterium compound-based MRS method in assessing tissue metabolokinetics 73. DMRS-based deuterium metabolic imaging has also been shown to be useful in detecting tumor cells 74. Recently, Laurie et al. proposed quantitative exchange-label turnover MRS, which can improve the sensitivity of the metabolic map and directly monitor cell metabolism *in vivo*. This approach is expected to be a new approach for exploring metabolic disorders in a wide range of human diseases 75. Based on the hypothesis that the conversion of amino acids in the presence of D_2_O leads to the production of deuterium-labeled amino acids 76-77, some studies have proved that the rate of protein synthesis can be estimated under the action of D_2_O 77-79. Herath et al. demonstrated that deuterium-based high-resolution mass spectrometry provides a useful method for the quantification of low levels of deuterium enrichment that is not limited to specific molecular classes; this method is expected to be useful for the study of metabolic flux of deuterium-labeled tracers 80.

## Development prospects

The exploration of the applications of heavy water provides novel insights and aids the development of new diagnostic and therapeutic strategies. Heavy water can be applied to the study of a variety of developmental processes, including cell development, metabolism, tissue homeostasis, drug resistance, and aging. As a nondestructive, noninvasive, and context-free imaging method, the combination of heavy water and Raman microscopy can be used to visualize the kinetics of protein synthesis, lipid production, and DNA metabolism in various model organisms at a low cost and without tissue bias. The latest developments in the applications of the heavy water labeling method indicate the possibility of real-time tracking of single cells, thus providing further understanding of the transport of single cells and making the *in vitro* study of single cells possible. This method will facilitate the comprehensive real-time molecular characterization and imaging of single cells *in vitro* to promote the understanding of single-cell biology. The newly developed classification strategy based on the C-D stretching vibration range avoids interference from other Raman bands owing to its higher sensitivity and thus saves time by negating the need to analyze large data sets. Heavy water provides a basis for the rapid clinical diagnosis and selection of appropriate antibiotics to treat bacterial infection. In particular, it provides a valuable method to facilitate the treatment and diagnosis of critical bacterial infections and can also be used to assay drug susceptibility. The recognition ability of heavy water to cancer stem cells with specific metabolic activities provides a new direction and visual angle for the diagnosis and treatment of clinical tumors. The combination of heavy water with Raman spectroscopy could be used to reveal the molecular components with biochemical significance through multivariable analysis, such as in the rapid identification of metabolically active drug-resistant cells or cancer stem cells with metabolic pattern changes after chemotherapy or in the exploration of the roles of such cells in chemotherapy failure, and could thus provide a direction for the development of personalized cancer therapy. Through the determination of heavy water concentration, data processing, and analysis framework, we can further promote the comparative study of extracellular vesicle uptake under different conditions or by different cell types, which may provide further guidance in the study of the role of different molecules, targeting and uptake of extracellular vesicles, and design of extracellular vesicle-based therapeutics. Heavy water provides data on different penetration depths and molecular skin effects to varying degrees, supporting the idea of multiple roles of heavy water in the skin as a convenient and inexpensive target.

As an emerging class of biomarker, heavy water has considerable advantages over traditional markers. Currently, several studies have partially elucidated the value of heavy water in medical research. However, the applications of heavy water in medical research need to be further explored, and heavy water is expected to play a more important role in the development of novel clinical management modalities.

## Abbreviations

D: heavy hydrogen
H_2_O: water
MIC-MA: minimum inhibitory concentration based on metabolic activity
O: oxygen
